# A case report of disseminated histoplasmosis and concurrent cryptococcal meningitis in a patient treated with ruxolitinib

**DOI:** 10.1186/s12879-019-3922-6

**Published:** 2019-03-27

**Authors:** Katya Prakash, Douglas Richman

**Affiliations:** 1 Division of Infectious Diseases, University of California San Diego, 9500 Gilman Drive, La Jolla, CA 92093-0711 USA; 2 VA San Diego Healthcare System, 3350 La Jolla Village Drive, San Diego, CA 92161 USA

**Keywords:** Ruxolitinib, JAKinhibitor, Immunosuppression, Cryptococcus, Histoplasmosis

## Abstract

**Background:**

Ruxolitinib is a highly potent janus kinase inhibitor that places its users at risk for various bacterial infections and viral reactivation. However new reports are also emerging that suggest greater immunosuppression and risk for fungal disease.

**Case presentation:**

We report the case of a 51 year-old veteran from Guam, treated with ruxolitinib for polycythemia vera, who developed disseminated histoplasmosis and concurrent cryptococcal meningitis.

**Conclusion:**

This case draws attention to the degree of immunosuppression that may be seen with this drug and the need for heightened vigilance for opportunistic infections in those treated with inhibitors of janus kinase/signal transducers and activators of transcription (JAK/STAT) such as ruxolitinib.

## Background

Ruxolitinib is a highly potent JAK1 and JAK2 inhibitor, FDA approved for the treatment of myelofibrosis and polycythemia vera. The JAK/STAT pathway is the principal signaling mechanism for numerous cytokines and growth factors. It is responsible for cell proliferation, differentiation, cell migration and apoptosis, which in turn are critical for efficient and successful hematopoiesis and immune development [1]. Many studies have attempted to fully elucidate the mechanism by which ruxolitinib impacts the immune system. Stuebig et al. investigated in vivo and in vitro effects of ruxolitinib on T cells and found that the drug impairs T cells proliferation by inducing apoptosis through an up regulation of p53 [2]. Studies also suggest that ruxolitinib strongly affects dendritic cell (DC) function. Monocytes differentiated in the presence of ruxolitinib did not develop any morphologic DC features, thought to be due to inhibition of GM-CSF and IL-4 signaling pathways [3]. Heine et al. also demonstrated that ruxolitinib affected the function and phenotype in preexisting DCs. With this effect on both T cell and DC function, one may predict risk for multiple viral, fungal and other opportunistic pathogens.

The phase 3 study of ruxolitinib (COMFORT II trial) [4] reported that reactivation of herpes zoster virus and tuberculosis were the predominant infections seen with ruxolitinib, as well as a trend towards higher rates of bacterial infections. Since that time, case reports have emerged, documenting the development of Pneumocystis *jiroveci* pneumonitis, toxoplasmosis retinitis, progressive multifocal leukoencephalopathy (PML), as well as cryptococcal meningoencephalitis and pulmonary cryptococcus in patients treated with ruxolitinib [5–9].

We report a case of concurrent disseminated Histoplasmosis and cryptococcal meningitis in a young patient from Guam on ruxolitinib that developed within months of starting the medication. The case highlights the high degree of immunosuppression that can be seen with JAK/STAT inhibitors.

## Case presentation

A 51 year-old male veteran presented with progressive lethargy, fevers and constant frontotemporal headache for past 3 weeks as well as 20 pound weight loss in past 6 months. Born in Guam, the patient had been stationed as part of the military in Texas, Arizona and Kansas. His medical history was notable for polycythemia vera (PCV) treated with ruxolitinib for 18 months. Three months before admission, he had recurrent mouth ulcers, followed by a dental root canal procedure complicated by ulcerative gingivitis, pulpitis and tooth erosions requiring antibiotics and multiple oral surgeries. All antimicrobials had been discontinued over a month prior to presentation.

On admission the patient was febrile to 103.5 °F, tachycardic, and saturating 95% on 2 l of oxygen by nasal cannula. Physical exam revealed somnolence, diminished breath sounds at the left lung base and diffuse abdominal tenderness. Neurologic exam identified no focal deficits. Initial laboratory studies (normal range) revealed hyponatremia to 125 (136–145) mmol/L and a creatinine elevation to 1.8 (0.67–1.17) mg/dL. He also had an elevated alkaline phosphatase of 208 (35–140) U/L and total bilirubin of 1.6 (< 1.2) mg/dL. White cell count was 8002 (4000-10,000) cells/mm^3^ with 74% polymorphonuclear cells and 13% lymphocytes. The C-reactive protein level was 3.89 (< 0.5) mg/dL and the erythrocyte sedimentation rate was 36 (< 30) mm/hr. Rapid HIV antibody testing, as well as HIV viral load, were negative.

**Fig. 1:**
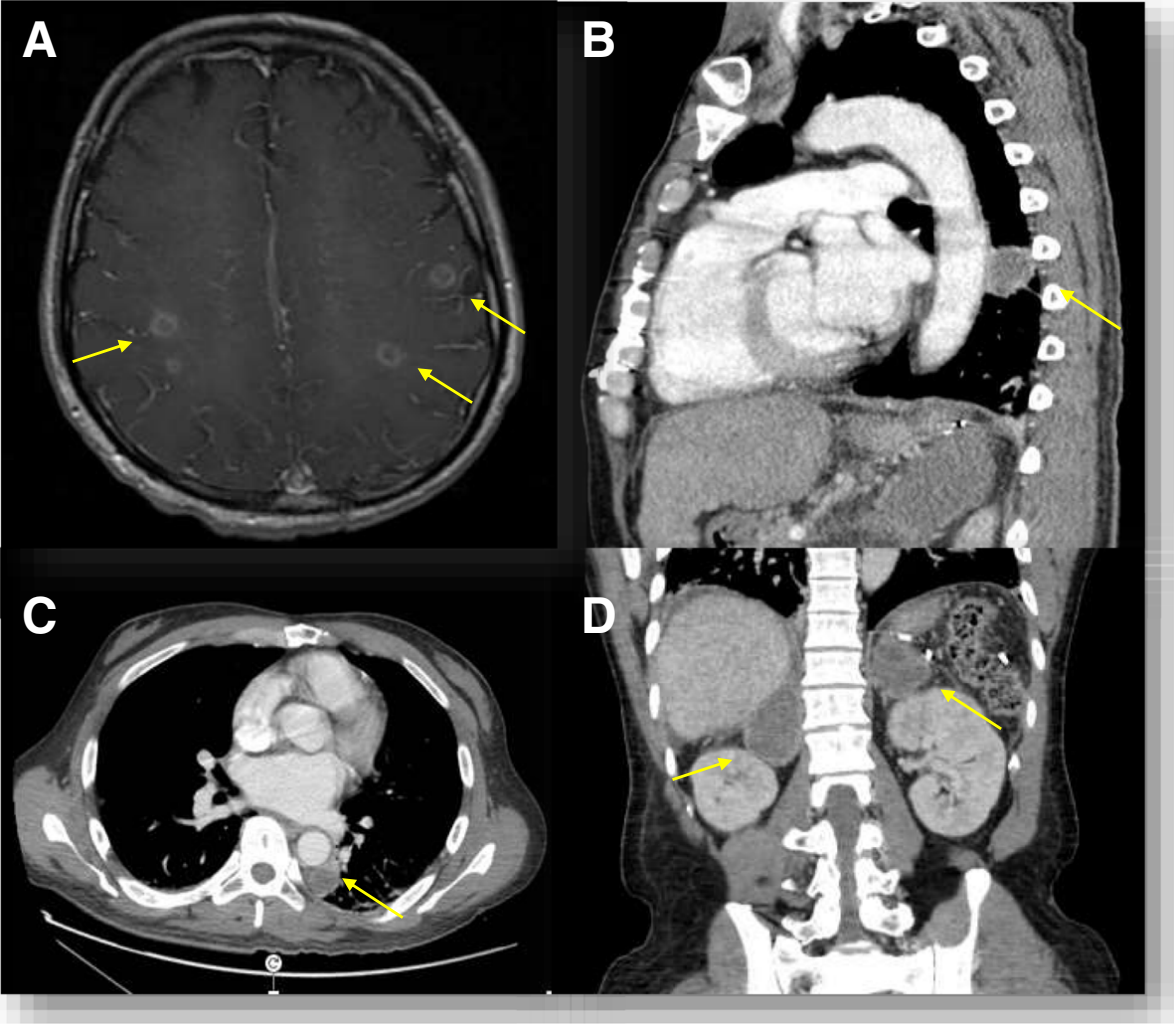
**a** T-1 weighted brain MRI with gadolinium showing innumerable rim enhancing lesions mostly at the gray-white junction. **b** and **c** CT thorax, abdomen and pelvis with contrast demonstrating a retrocardiac rim enhancing mass, measuring 2.7 cm and (**d**) masses infiltrating adrenal glands bilaterally, left larger than right measuring 8 cm

The brain MRI revealed innumerable rim enhancing lesions at the gray-white junction consistent with pyogenic abscesses secondary to hematogenous infection (Fig. 1a). A lumbar puncture revealed 10 mononuclear cells and 9 polymorphonuclear cells/ml CSF. Glucose was 27 (40–70) mg/dL and protein was 72 (15–45) mg/dL. Vancomycin, ceftriaxone and metronidazole were initiated empirically.

**Fig. 2:**
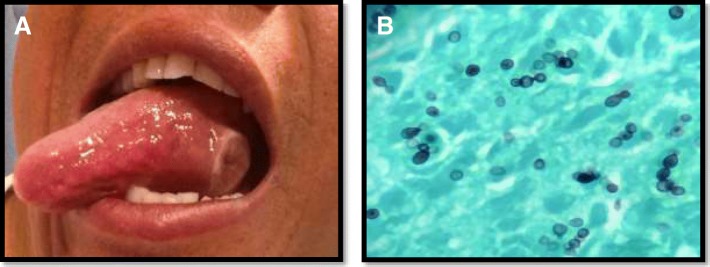
**a** 2 cm painful yellow ulcerative tongue lesion that first appeared three months after initiating ruxolitinib. **b** GMS staining revealing small budding yeast measuring 2–4 μm

The patient subsequently underwent chest (Fig. 2b and c) and abdominal CT examinations (Fig. 2d) to evaluate diminished breath sounds and abdominal tenderness. A retrocardiac mass was seen measuring 2.7 cm as well as bilaterally enlarged adrenal glands consistent with infiltrative infection or neoplasm.

CSF cultures grew no bacteria; however, cryptococcal antigen was detected with titer of 1:> 256 in CSF and 1:128 in the serum. Fungal CSF cultures grew *Cryptococcus neoformans*. An adrenal biopsy performed by interventional radiology revealed numerous fungal organisms on histopathology. Gomorri methenamine silver (GMS) and periodic acid-Schiff (PAS) stains highlighted budding yeast forms within macrophages, most consistent with histoplasmosis. *Histoplasma capsulatum* subsequently grew in fungal blood cultures. Histoplasma antigen was 11.9 (< 0.5) ng/mL in urine, 8.46 (< 0.4) ng/mL in the serum and 1.86 (< 0.4) ng/mL in CSF. Cryptococcal susceptibilities ultimately returned with an MIC of < 0.03 μg/ml to isavuconazole and 0.25 μg/ml to posaconazole; Histoplasma susceptibilities were < 0.03 μg/ml to both isavuconazole and posaconazole. The patient was diagnosed with concurrent cryptococcal meningitis as well as disseminated histoplasmosis.

Amphotericin infusion as Ambisome at 5 mg/kg every 48 h with flucytosine 1 g q6hrs were initiated for treatment of both identified fungal organisms with improvement of symptoms. Given limited evidence of the successful use of the newer azoles, posaconazole [10–13] and isavuconazole [14–17] for CNS disease, the patient was continued on amphotericin infusions for three months and transitioned to 372 mg isavuconazole daily when renal toxicity was noted with Ambisome. A follow up MRI at that time demonstrated diminishing rim-enhancing lesions. The retrocardiac mass was smaller in size on repeat imaging; however, the appearance of the adrenal glands remained unchanged. Cryptococcal antigen titers were 1:16 in serum and 1:8 in CSF. Histoplasmosis antigen in the urine was 0.83 (< 0.5) ng/mL and was no longer detected in the serum. A biopsy of the brain lesions was not performed; however, we hypothesized that the brain lesions were caused by hematogenous spread of histoplasmosis to the gray-white junction with resulting granuloma formation. At the time this case report was written, the patient was still being treated with isavuconazole.

Of note, once the patient was diagnosed with the two fungal infections, ruxolitinib was discontinued. Given the severity of his presentation, his primary oncologist believed that a re-challenge with ruxolitinib was contraindicated.

After further discussion with the patient, he had recollected a 2 cm ulcerative, painful tongue mass (Fig. 2a) that first appeared three months after initiating ruxolitinib. This had been biopsied in the past and was not malignant, but had not been evaluated with fungal culture. Retrospective review of the pathology slides was suggestive of histoplasmosis, with small budding yeast forms noted within granulomas on GMS stain (Fig. 2b).

## Discussion and conclusion

To the best of our knowledge this is the first case of both disseminated histoplasmosis and cryptococcal meningitis. This case draws attention to three key factors. The first is the degree of immunosuppression seen with ruxolitinib and the increasing number of reported fungal cases associated with the drug [5–8]. The second, which was noted on initial phase III trials of ruxolitinib, but must be emphasized, is that the adverse effects from immunosuppression may occur at various intervals after the initiation of ruxolitinib. And third, there is a scarcity of clinical trials and pharmacokinetic studies on the newer azoles that can support their use as step down therapy in severe CNS infections with these fungi.

Our case stresses the need for heightened vigilance for opportunistic infections complicating treatment with ruxolitinib or any JAK inhibitor, of which several appear to be in phase II trials at this time. Since ruxolitinib came to the market, multiple case reports have surfaced detailing its various infectious complications. A review published in 2018 by Dioverti et al., identified 32 cases of opportunistic infections in the setting of ruxolitinib [18]. While the majority of cases reported were either reactivations of tuberculosis (34%) or hepatitis B virus (9%), several fungal infections were also identified. Pulmonary as well as extrapulmonary cryptococcus was the most frequently reported fungus. Several cases of pneumocystis pneumonia and one case of rhino-orbital mucormycosis were also described. Histoplasmosis infections have not been reported previously.

It is also important to recognize that these infections can occur at any point in the course of taking the JAK inhibitor. In our patient, the first symptom, i.e. the tongue lesion, a likely oral manifestation of histoplasmosis, appeared within 3 months of initiating the drug. In reviewing the literature, immunosuppression associated with ruxolitinib was not time dependent occurring anywhere between 1 and over 100 weeks after initiation of the drug [4, 18]. Whether its effect is dose-dependent is still controversial. In the majority of cases ruxolitinib had been discontinued once a major infection was identified; however, for several cases not involving fungal infections, dose reductions were implemented [18]. In the past few years, there has been debate regarding whether the degree of immunosuppression seen with JAK inhibitors mandates initiation of prophylaxis [19, 20]. At this time, we do not believe that this is warranted given the relative infrequency of fungal complications recognized. However, a more proactive pre-emptive approach, that includes routine screening for those with high risk exposures may be prudent.

Although CNS histoplasmosis is still relatively rare [17, 21], other fungal infections involving the central nervous system are not, and azole resistance is on the rise [22]. In addition, azole toxicities and drug-drug interactions limit their use. Azoles are inhibitors and substrates of the CYP2C19, 2C9 and 3A4 enzymes and thus interact with other drugs that use these same CYP450 enzymes for metabolism (notably antiretrovirals, immunosuppressive drugs and cardiovascular medications) [23, 24]. Further clinical trials are needed to evaluate newer agents and their success in infections involving the nervous system.

## Abbreviations

CNS: Central nervous system
CSF: Cerebrospinal fluid
DC: Dendritic cell
FDA: Food and Drug Administration
GMS: Gomori methenamine-silver
HIV: Human immunodeficiency virus
JAK/STAT: Janus kinase/signal transducers and activators of transcription
PAS: Periodic acid-Schiff
PCV: Polycythemia vera
PML: Progressive multifocal leukoencephalopathy
